# Health Care System Collaboration to Address Chronic Diseases: A Nationwide Snapshot From State Public Health Practitioners

**DOI:** 10.5888/pcd11.140075

**Published:** 2014-09-04

**Authors:** Lindsay Elliott, Timothy D. McBride, Peg Allen, Rebekah R. Jacob, Ellen Jones, Jon Kerner, Ross C. Brownson

**Affiliations:** Author Affiliations: Timothy D. McBride, Peg Allen, Rebekah Jacob, Ross C. Brownson, Brown School, Washington University in St Louis, St Louis, Missouri; Ellen Jones, University of Mississippi Medical Center, Jackson, Mississippi; Jon Kerner, Canadian Partnership Against Cancer, Toronto, Ontario.

## Abstract

**Introduction:**

Until recently, health care systems in the United States often lacked a unified approach to prevent and manage chronic disease. Recent efforts have been made to close this gap through various calls for increased collaboration between public health and health care systems to better coordinate provision of services and programs. Currently, the extent to which the public health workforce has responded is relatively unknown. The objective of this study is to explore health care system collaboration efforts and activities among a population-based sample of state public health practitioners.

**Methods:**

During spring 2013, a national survey was administered to state-level chronic disease public health practitioners. Respondents were asked to indicate whether or not they collaborate with health care systems. Those who reported “yes” were asked to indicate all topic areas in which they collaborate and provide qualitative examples of their collaborative work.

**Results:**

A total of 759 respondents (84%) reported collaboration. Common topics of collaboration activities were tobacco, cardiovascular health, and cancer screening. More client-oriented interventions than system-wide interventions were found in the qualitative examples provided. Respondents who collaborated were also more likely to use the Community Guide, use evidence-based decision making, and work in program areas that involved secondary, rather than primary, prevention.

**Conclusion:**

The study findings indicate a need for greater guidance on collaboration efforts that involve system-wide and cross-system interventions. Tools such as the Community Guide and evidence-based training courses may be useful in providing such guidance.

## Introduction

Effective prevention and management of chronic conditions is a high priority — chronic disease affects nearly 1 in 2 adults in the United States and accounts for 75% of our nation’s health care costs (1,2). Efforts to reduce the burden of chronic disease have been fragmented, with different health care systems working independently to achieve outcomes (3).

Increased collaboration among different health care systems to prevent and manage chronic diseases has been recently prioritized (3–6). In 2012, for example, the Institute of Medicine (IOM) published a national report, *Primary Care and Public Health: Exploring Integration to Improve Population Health,* which recognized need and identified opportunities for health care to improve population health (3). The Patient Protection and Affordable Care Act (ACA) emerged in the IOM’s report as a key vehicle for health systems to achieve increased collaboration.

The Centers for Disease Control and Prevention (CDC) and the Public Health Accreditation Board (PHAB) have reinforced the vision for more unified chronic disease approaches across health care systems. Two of CDC’s 4 key chronic disease practice domains call on public health to improve the services provided by health care systems (4). Health care system collaboration is now required by PHAB for health departments’ accreditation, and the movement toward collaborative chronic care continues to grow (5).

However, despite the growing emphasis on these issues, few data are available on how the public health workforce has responded to the call for greater collaboration. The objective of our study is to bridge this gap by using a mixed methods study with 2 goals: 1) to explore the patterns and correlates of public health’s collaboration with health care systems from the perspective of state public health practitioners, and 2) to compare practitioners’ collaborations with those recommended by CDC.

## Methods

During spring 2013, a national online survey was administered to state-level public health practitioners whose primary work was preventing and managing chronic diseases. Human subject approval was obtained from the Institutional Review Board of Washington University in St Louis.

State health department program managers and staff from all 50 states and the District of Columbia were invited to participate in the survey if they primarily worked in program areas of cancer, tobacco, physical activity, nutrition, obesity prevention, diabetes prevention, and cardiovascular health. Eligible invitees were identified by searching state health department websites and member lists obtained from partner organizations. Eligibility of invitees was verified by telephone as needed. A total of 1,169 eligible participants were invited to complete the survey through a personalized e-mail invitation. Respondents were offered a $20 Amazon.com gift card on completion of the survey.

Previous research and 5 rounds of advisory input from an international team were used to construct the initial survey instrument (7–9). The survey was refined through cognitive response testing with 11 former chronic disease practitioners, and a reliability test–retest sample of 75 current state-level chronic disease practitioners was used to establish internal consistency among most of the 68 items included in the final survey (Cronbach’s α ≥ .70). The final 15-minute survey was programmed into Qualtrics survey software (Qualtrics, LLC) for online administration. Further description of survey development and data collection are published elsewhere (9), and a copy of the full questionnaire is available on request.

### Measures

Although the survey’s content was designed to explore a multitude of purposes, our study focused on characteristics of state health department practitioners and their program or work unit’s collaborations with health care systems, their personal use of evidence-based decision making (EBDM), and their personal use of the US Preventive Services Task Force’s Community Guide. Items used to assess these concepts are described below.

Characteristics of state health department practitioners were assessed through basic demographic questions, which included items such as age, sex, years worked in public health, and the main program area in which respondents worked. All respondents were asked to indicate their personal use of EBDM within their work by using a single item, with a 7-point Likert scale ranging from “strongly disagree” to “strongly agree.” The concept of EBDM was defined for respondents as “prioritizing issues and implementing interventions based on sound science combined with community engagement, sound management, and evaluation” (10).

Respondents were asked to report their use of the Community Guide. Response options included “yes, often,” “yes, sometimes,” “no,” and “I'm not familiar with the Community Guide.”

**Figure 1:**
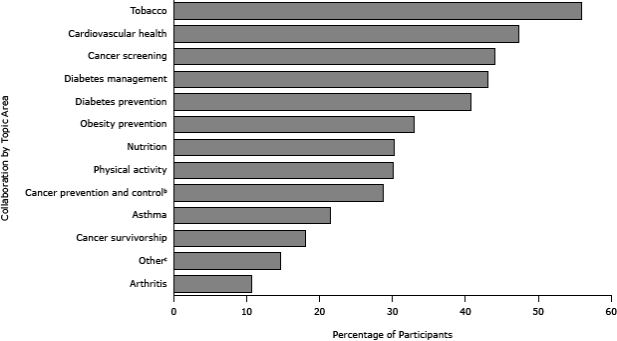
Self-reported topic areas for health care system collaboration (N = 759). Participants in a 2013 national survey of state health department chronic disease prevention staff who reported collaboration with health care systems were asked to indicate all topic areas of collaboration from a provided list. Percentages total more than 100% because participants could choose all topic areas that applied. Cancer prevention and control programs do not include cancer screening. “Other” areas commonly self-reported were maternal–child health, breast-feeding, cancer registry, and adolescent health. **Table:** 

Health care system collaboration, the primary outcome for our study, was assessed through 3 items. Respondents were asked, “Does your program or work unit collaborate with health care systems that include hospitals, outpatient clinics, and/or Federally-qualified health centers?” Response options included “yes,” “no,” and “I’m not sure.” Respondents who selected “yes” were asked to indicate topic areas of collaboration activities from a provided list of options (Figure 1). Additionally, respondents who reported collaboration were asked to type an example of their collaborative work into a text box.

Topic Area of Collaboration Activity	%^a^
Tobacco	55.9
Cardiovascular health	47.3
Cancer screening	44.0
Diabetes management	43.1
Diabetes prevention	40.7
Obesity prevention	33.0
Nutrition	30.2
Physical activity	30.1
Cancer prevention and control	28.7
Asthma	21.5
Cancer survivorship	18.0
Other	14.7
Arthritis	10.7

### Data analysis

Descriptive and multivariate analyses were conducted using IBM SPSS Statistics 21 to explore frequencies and associations with health care system collaboration. “Health care system collaboration,” “use of EBDM,” and “use of Community Guide” were recoded into binary variables. Significance was set at *P* < .05.

For multivariate analyses, differences in health care system collaboration were examined through bivariate analyses that used both survey data and state characteristics (11–13), and the bivariate results were used to construct logistic regression models. Variables with moderate to high proportions of shared variance were not included in the model to reduce possible effects of multicollinearity (Pearson’s *r* > .20). The final model included “program area,” “use of EBDM,” and “use of Community Guide” and met all model assumptions.

**Figure 2:**
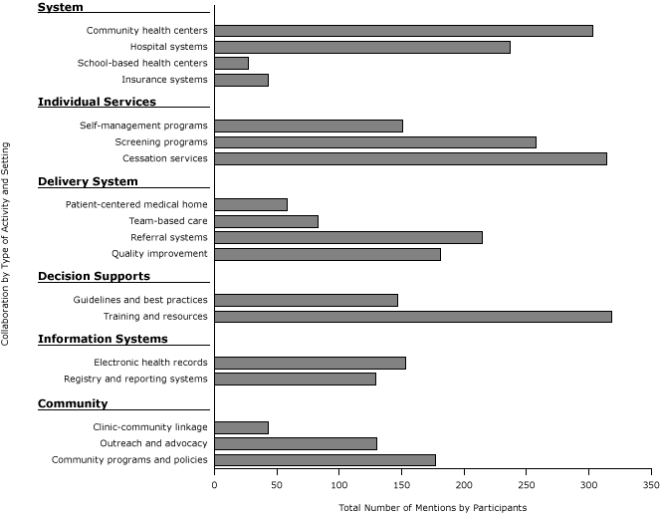
Collaboration examples by common settings and activities, derived from qualitative data (N = 642). Participants in a 2013 national survey of state health department chronic disease prevention staff who reported collaboration with health care systems were asked to provide an example of their collaborative work. **Table:** 

Qualitative methods were used to code common themes among respondents’ health care collaboration examples. Common themes were grouped into 14 categories based on the Chronic Care Model, a 6-component framework that improves chronic care and associated health outcomes (Figure 2) (14). Two authors (L.E., R.J.) independently coded the 642 collaboration examples by using QSR NVivo 10 software (QSR International Pty Ltd) with high average inter-rater agreement (κ = .86).

Type of Setting or Activity	n
Community health centers	303
Hospital systems	237
School-based health centers	27
Insurance systems	43
Self-management programs	151
Screening programs	258
Cessation services	314
Patient-centered medical home	58
Team-based care	83
Referral systems	215
Quality improvement	181
Guidelines and best practices	147
Training and resources	318
Electronic health records	153
Registry and reporting systems	129
Clinic-community linkage	43
Outreach and advocacy	130
Community programs and policies	177

## Results

We asked respondents to answer yes or no to the health care system collaboration survey item (Table 1). Of the 759 (84.0%) respondents who indicated collaboration with health care systems, respondents with more years worked in their specific health agency, their current position, and generally in public health were more likely to report collaboration (*P* = .006, *P* = .02, *P* = .01, respectively). In contrast, collaboration did not differ by respondents’ sex, age group, degree type (clinical vs nonclinical), or educational attainment.

**Table 1:** Participant and State Characteristics by Health Care Collaboration in a National Sample of US State Health Department Staff in Chronic Disease Prevention, 2013 (N = 904)

Characteristics	Total Sample (N = 904)	Did Not Report Collaboration (N = 144)	Reported Collaboration (N = 759)^a^	*P* Value^b^
	n (%)			
Program area				
Cancer prevention and control	145 (16.1)	6 (4.2)	139 (18.3)	<.001
Tobacco control	161 (17.8)	30 (20.8)	131 (17.3)	
Obesity, physical activity, nutrition	132 (14.6)	45 (31.3)	87 (11.5)	
Cardiovascular health and diabetes	151 (16.7)	6 (4.2)	145 (19.1)	
Multiple areas^c^	255 (28.2)	38 (26.4)	217 (28.6)	
Other areas^d^	59 (6.5)	19 (13.2)	40 (5.3)	
Sex				
Female	726 (80.4)	108 (75.0)	618 (81.4)	.08
Male	177 (19.6)	36 (25.0)	141 (18.6)	
Age, y				
20–39	277 (30.8)	52 (36.4)	225 (29.8)	.07
40–49	248 (27.6)	46 (32.2)	202 (26.8)	
50–59	246 (27.3)	29 (20.3)	217 (28.7)	
≥60	127 (14.1)	16 (11.2)	111 (14.7)	
Use of Community Guide				
Often/Sometimes	718 (80.0)	92 (63.9)	626 (83.0)	<.001
No/Don’t know	180 (20.0)	52 (36.1)	128 (17.0)	
Use of EBDM^e^ in work				
Agree	781 (87.7)	114 (80.9)	667 (88.9)	.007
Do not agree	110 (12.3)	27 (19.9)	83 (11.1)	
Educational level				
Master’s degree or higher	632 (70.0)	95 (66.0)	537 (70.8)	.25
No master’s degree	271 (30.0)	49 (34.0)	222 (29.2)	
Degree type				
Clinical degree^f^	177 (19.8)	26 (18.1)	151 (20.1)	.57
Nonclinical degree	717 (80.2)	118 (81.9)	599 (79.9)	
Years worked at agency, mean (SD)	9.9 (7.9)	8.3 (7.0)	10.2 (8.0)	.006
Years worked in position, mean (SD)	4.9 (4.9)	4.2 (3.9)	5.1 (5.0)	.02
Years worked in public health, mean (SD)	14.7 (9.2)	12.9 (8.8)	15.0 (9.3)	.01
Chronic disease funding per capita^g^, mean (SD)	1.5 (1.2)	1.3 (1.1)	1.5 (1.2)	.04
Total cancer mortality rate per 1000^h^, mean (SD)	14.1 (14.1)	18.2 (17.5)	13.3 (13.3)	.002
Percentage of state uninsured (%)^i^ , mean (SD)	14.4 (4.1)	15.1 (4.3)	14.2 (4.1)	.02

Abbreviations: EBDM, evidence-based decision making; SD, standard deviation.

a Because of missing data, not all categories total 144 and 759. Percentages represent valid nonmissing cases.

b *P* values for continuous variables were calculated by *t* tests, and χ^2^ tests were conducted for binary variables to test significance.

c Multiple areas included generalists or practitioners whose primary work spanned several chronic disease program areas (eg, epidemiologists, chronic disease directors).

d Other areas included arthritis, school health, oral health, and other, less commonly mentioned areas.

e EBDM is defined as “prioritizing issues and implementing interventions based on sound science combined with community engagement, sound management, and evaluation” (10).

f Clinical degrees include doctor of medicine, doctor of osteopathic medicine, doctor of dental surgery, registered dietitian, certified diabetes educator, and all nursing degrees (registered nurse, licensed practical nurse, bachelor of science in nursing, advanced practice registered nursing, or “other nursing”).

g Data from Centers for Disease Control and Prevention, *Justification of Estimates for Appropriations Committees*. Totaled from 4 chronic disease categories: breast and cervical cancer, tobacco, comprehensive cancer, diabetes prevention (11).

h Data from National Cancer Institute and Centers for Disease Control and Prevention, *State Cancer Profiles* (12).

i Data from US Census Bureau American Community Survey, *2010 American Community Survey 1-Year Estimates* (13).

A higher percentage (83%) of respondents who reported collaboration reported use of EBDM in their work and use of the Community Guide compared with those who did not report collaboration (*P* = .007 and *P* < .001, respectively). Health system collaboration was associated with the main program area in which respondents worked and was more often reported among those working across multiple program areas (28.6%), in cardiovascular health and diabetes (19.1%), or in cancer prevention and control (18.3%) compared with other areas (*P* < .001).

Bivariate analyses also indicated significant differences in health care collaboration by state characteristics. Respondents from states with higher chronic disease funding dollars per capita, lower cancer mortality rates, and lower percentage of population uninsured were more likely to report collaboration (*P* = .04, *P* = .002, *P* = .02, respectively).

Self-reported topics of collaboration activities from the 759 respondents who reported collaboration are displayed in Figure 1. Tobacco cessation was the most commonly reported topic area of collaboration (55.9%), followed by cardiovascular health (47.2%), cancer screening (44.0%), and diabetes management (43.1%). Common “other” responses included maternal-child health, breastfeeding, and cancer registry.

We grouped the 642 qualitative examples of self-reported collaboration by type of collaborative activity and health system settings (Figure 2). Respondents most frequently reported collaborative work with community health centers (47.2%) and hospital systems (36.9%). Commonly mentioned collaborative activities were training providers and providing resources such as toolkits (49.5%), tobacco cessation services such as quitline referrals and support for cessation programs (48.9%), and general cancer screening services (40.2%). The least common collaboration activities mentioned included clinic–community linkages (6.7%), support for Patient-Centered Medical Homes (9.0%), and promotion of team-based care (eg, promotion of patient navigators) (12.9%). We list respondents’ comments about these and other collaborations (Box).

**Box. Health Care Collaboration Among State Health Department Chronic Disease Prevention Staff From All 50 US States That Reported Collaboration With Health Care Systems, Spring 2013 (N = 642)**

**Table:** 

Activity	Collaboration Examples
Individual services	“The program reviews the Certifications of the 32 Diabetes Self-Management Training (DSMT) Sites, collects sites’ CQI objectives, and DSMT participant goals (set and accomplished). Oversees and administers the New Instructor Program for all DSMT instructors in the State.”
Delivery system	“We have implemented a program that assists primary care health care practices to implement components of the chronic care model to become NCQA-recognized patient-centered medical homes.”
	“[Changed] EHR so that a fully electronic process for identifying, offering assistance, and referring those that wanted assistance to quit tobacco was developed. Further, the [cessation] counselor treating the patient electronically sends a follow up report back to the referring provider.”
Decision supports	“Health systems change and infrastructure building to ensure patients are screened for CRC. Examples include office policy development, building EHR cancer registries, and developing patient reminders.”
	“[State] set up a Chronic Disease Collaborative that involves members across various sectors including health care systems to address common goals, objectives, and strategies. This opportunity allows members to collaborate and share information and resources.”
Information systems	“We work with the largest health insurance provider to implement a free clinical information system. This free Web-based system . . . allows the providers to monitor [risk factors] . . . see at a glance missed care opportunities and can generate letters to send to patients to encourage them to call the office for an appointment. We currently have over 70% of the state providers enrolled in the system.”
	“We are currently working on a collaborative effort to build a network of electronic records access to help improve the surveillance aspects of chronic disease in [state] while linking this with Medicaid. There is a lot of coordination happening to adopt this in our state.”
Community	“A centralized referral system has been implemented with patient navigation services to assist the patients referred from the practices to [12 community evidence-based lifestyle and disease management programs].”

Abbreviations: CQI, continuous quality improvement; CRC, colorectal cancer; EHR, electronic health record; NCQA, National Committee for Quality Assurance.

Accounting for multivariate associations, participants who used the Community Guide were 2.6 times as likely to report collaboration with health care systems as those who did not use the Community Guide (Table 2) (*P* < .001). Similarly, participants who used EBDM were twice as likely to report collaboration as those who did not use EBDM (*P* = .01). The association between collaboration and the main program area in which respondents worked remained robust after adjusting for use of EBDM and the Community Guide. Compared with those working in obesity, physical activity, or nutrition, participants working in cancer prevention and control were 11.9 times as likely to report collaboration (*P* < .001), and those working in cardiovascular health and diabetes were 14.5 times as likely to report collaboration with health systems (*P* < .001). Although bivariate results indicated significant associations between state characteristics and health care system collaboration, practitioners’ likelihood of collaborating was not significantly influenced by state characteristics after adjusting for clustering effects by state membership.

**Table 2:** Health Care Collaboration in a National 2013 Sample of State Health Department Chronic Disease Prevention Staff (N = 904)^a^

Characteristics	OR (95% CI)	*P* Value^b^
**Program area**		
Obesity, physical activity, nutrition	1 [Reference]	—
Tobacco control	2.3 (1.3–3.9)	.004
Cancer prevention and control	11.9 (4.8–29.6)	<.001
Cardiovascular health and diabetes	14.5 (5.8–36.0)	<.001
Multiple areas	3.1 (1.8–5.1)	<.001
Other areas	1.2 (0.6–2.3)	.67
**Use of Community Guide**		
No/Don’t know^c^	1 [Reference]	—
Yes	2.6 (1.7–4.0)	<.001
**Use of EBDM** c **in work**		
Do not agree	1 [Reference]	—
Agree	2.0 (1.2–3.3)	.01

Abbreviations: OR, odds ratio; CI, confidence interval; —, no P values for reference categories; EBDM, evidence-based decision making.

a Multivariate odds ratios for health care collaboration likelihood are adjusted for program area, use of evidence-based decision making, and use of the Community Guide.

b Logistic regression model used to determine *P* values of independent variables based on the Wald test for significance.

c EBDM is defined as “prioritizing issues and implementing interventions based on sound science combined with community engagement, sound management, and evaluation” (10).

## Discussion

The 2012 IOM report highlighted a key challenge: few examples of successful collaborative care between public health and health care systems on a large scale are known (3). Our study aimed to address this challenge by collecting and analyzing a nationwide sample of state health department practitioners’ collaborative efforts with health care systems to prevent and manage chronic disease. Our findings indicate that the public health workforce has responded to the call to action — most state health department practitioners in chronic disease prevention report that their work unit or program area collaborates with health care systems.

Standards set by CDC and PHAB may promote collaborations between public health and health care systems. Of the 4 practice domains in CDC’s National Center for Chronic Disease Prevention and Health Promotion, 2 require public health’s collaboration with health care systems. Domain 3 states that public health should improve the quality and accessibility of preventive services offered by health systems through use of health information technology, evidence-based chronic care delivery, and other interventions that align with the Community Guide (4). Domain 4, community-clinical linkages, encourages public health to facilitate cross-system referrals between clinics and communities to ensure that people who have or are at risk for chronic diseases have access to CDC-recommended programs and services to help prevent or manage their chronic conditions (4).

The PHAB echoes CDC’s recommendations. The accreditation of health departments has been referred to as one of the most important initiatives in transforming the performance and practice of public health practitioners (15). Twelve domains outline required actions for state health department accreditation. Domain 7 specifically requires health departments to link with health care systems through collaborative processes to improve health care systems’ capacity and provision of preventive services (5).

In general, respondents mentioned types of collaboration activities recommended by CDC and PHAB. However, findings from this study indicate that state health departments may emphasize collaborations that focus on client-oriented services over changes to delivery and information systems. For example, respondents mentioned tobacco cessation and cancer screening services more frequently than electronic health records (EHRs) and referral systems.

Similar findings were published in a recent report of CDC’s colorectal cancer screening programs. Hannon and colleagues found that state health departments funded to increase screening among underserved populations were more likely to use client-oriented interventions (eg, small media) than interventions involving system-wide procedures such as provider reminder and recall systems (16). They also found that most grantees (96%) worked with health systems on small media interventions, yet only 32% of grantees reported using automated provider reminder systems (16). All of the 32% who successfully implemented provider reminders reported using EHRs. Grantees also noted difficulty in working with EHRs as a barrier to implementation of CDC-recommended interventions (16).

Further exploration of the relationship between public health’s implementation of evidence-based interventions and health care systems’ EHR capacity may be useful. Although use of EHRs increases the number of preventive services offered in clinical settings and improves health outcomes, they are underutilized (17–21). Maylahn and colleagues recently recommended collaborations involving EHRs to help public health and health care systems achieve common goals (22). More research is needed to identify collaborations that increase EHR capacity and implementation of recommended interventions.

Our study found that public health practitioners who reported use of the Community Guide and use of EBDM were significantly more likely to report collaboration with health care systems. Although the direction of the relationship cannot be determined from these cross-sectional data, our findings suggest that CDC’s promotion of the Community Guide through grant funding and practice domains is valuable as a potential guide to collaborative work.

A twofold increase in health care system collaboration was found among practitioners who used EBDM, which warrants exploring how training programs that teach EBDM competencies might influence collaboration activities. Courses in evidence-based public health teach practitioners a range of skills that have been shown to promote use of EBDM (23,24). Such courses may be used as training resources for collaborative activities.

Although no interactions were found between respondents’ program area, use of the Community Guide, and use of EBDM, we found differences in respondents’ likelihood to collaborate with health systems based on the main program area in which individuals worked. Program areas with more focus on secondary prevention (eg, cancer, tobacco cessation, cardiovascular health and diabetes) were more likely to collaborate across sectors. Some of these findings were expected. The Community Guide has more systematic guidance on clinically focused interventions for practitioners who work in secondary prevention compared with obesity, physical activity, and nutrition (25). More work is needed to identify effective interventions and define how primary prevention programs can strengthen cross-sector collaborations.

### Recommendations

Findings from this study support the need for better guidance on collaborations that support health system infrastructure, and for increased collaboration guidance for additional chronic disease program areas. Although we found that most state health departments collaborate with health care systems, much work remains to be done. Patients receive only about half of the recommended clinical preventive services in primary care settings (16,26,27). Even with guidance from CDC and PHAB on evidence-based collaborative approaches, barriers inhibit state health departments’ use of such approaches. Lack of funding remains a common barrier (7,28). A key purpose of the IOM report was to identify potential funding avenues for collaboration. The IOM highlighted the ACA as a pathway for funding. Although Title IV of the ACA contains the most direct public health prevention funding opportunities, several ACA provisions, such as Patient-Centered Medical Homes and Accountable Care Organizations, allow for public health to invest in clinical services that promote more comprehensive chronic care (29). With combined efforts and leveraged resources, public health can work with health systems through these provisions to achieve common goals. In general, more research is needed to understand how state health departments use opportunities provided through the ACA to collaborate.

Results of collaborative work may guide future US collaborations. A scoping review from Canada on public health and health care collaboration validates benefits that include reduced hospital and emergency department use, improved chronic care delivery, increased identification of people at risk for chronic disease, and increased access to care (30). Such examples show that collaboration is both feasible and economically viable.

Because the survey was self-reported, we were unable to capture the quality and scope of responses and collaboration activities. For example, respondents were asked to report the collaboration of their program area or work unit, which makes it difficult to assess their personal involvement in collaborations. Self-reported survey data also make it difficult to delineate respondents’ primary program area; some between program areas may overlap, depending on different state health department structures. Respondents were not given a thorough definition of “health care system collaboration,” which may have caused interpretation inconsistencies among respondents. Lastly, the directionality of associations found cannot be determined because of the cross-sectional study design. Despite these limitations, our study contributes to the literature by identifying achievements and gaps in collaborations reported by a nationwide sample of public health practitioners and by providing next steps for collaboration guidance.

The increase in chronic diseases and the emphasis on collaboration from CDC and PHAB increase the likelihood that cross-sector efforts to prevent and manage chronic disease will grow. Systematic guidance is needed to identify collaboration activities that yield high public health impact and improved delivery systems for chronic care. Next steps may include comparisons of different types of cross-sector collaborations and their subsequent impact on system changes. Evaluation is also needed to better prioritize which types of collaboration activities should be the main focus of state health departments. Further research to fill the gaps identified by this study may help ensure that future collaborations result in improved systems, better care, and an overall reduced burden of chronic disease.
